# Beyond peak accuracy: a stability-centric framework for reliable multimodal student engagement assessment

**DOI:** 10.1038/s41598-025-31215-7

**Published:** 2026-01-02

**Authors:** Ismail Said Almuniri, Hitham Alhussian, Norshakirah Aziz, Sallam O. F. Khairy, AlWaleed Sulaiman AlAbri, Zaid Fawaz Jarallah, Saidu Yahaya, Shamsuddeen Adamu

**Affiliations:** 1 Department of Computing, Universiti Teknologi PETRONAS, Seri Iskandar, Malaysia; 2 Department of Information Systems, College of Economics, Management and Information Systems, University of Nizwa, Nizwa, Oman; 3 Ahmadu Bello University, Zaria, Nigeria; 4 Computer Science Department, College of Education for Pure Science, University of Mosul, Mosul, Iraq

**Keywords:** Multimodal learning analytics, Student engagement assessment, Temporal data augmentation, Ensemble learning, Interpretability, Educational data mining, Computational biology and bioinformatics, Mathematics and computing

## Abstract

Accurate assessment of student engagement is central to technology-enhanced learning, yet existing models remain constrained by class imbalance, instability across data splits, and limited interpretability. This study introduces a multimodal engagement assessment framework that addresses these issues through three complementary strategies: (1) class-aware loss functions to alleviate class imbalance, (2) temporal data augmentation and heterogeneous ensembling to enhance model stability, and (3) SHAP-based analysis of the most stable component for reliable interpretability. Reliability was established through repeated cross-validation with multiple seeds across seven deep learning architectures and the proposed ensemble. The framework established a mean accuracy of 0.901 ± 0.043 and a mean macro F1 of 0.847 ± 0.068, surpassing baselines such as ResNet (Accuracy = 0.917), Inception (Macro F1 = 0.862), and LightGBM (Accuracy = 0.922). Ablation studies highlighted temporal augmentation and ensemble diversity as key contributors, while sensitivity analyses confirmed robustness with variance consistently below 0.07 across seeds and folds. Efficiency profiling established MCNN and TimeCNN as the optimal deployment architecture, combining near-optimal accuracy with superior computational efficiency. SHAP-based interpretation was extended to provide feature-level and class-wise attribution, revealing consistent relationships between predictions and behavioral or cognitive cues. Overall, the study demonstrates that balanced evaluation and ensemble stability are essential for reliable engagement assessment.

## Introduction

Student engagement is widely recognized as essential for academic success, positive learning environments, and overall educational quality^1^. It functions as a multifaceted construct, encompassing behavioral, emotional, and cognitive dimensions, that directly influences learning outcomes and student satisfaction^1^. Accurate assessment of engagement allows educators to identify disengaged students, facilitate personalized learning experiences, and adapt instructional strategies to maximize effectiveness^2^. Furthermore, it provides invaluable data for educational researchers developing innovative pedagogical approaches and technologies^3^.

Traditional methods for assessing student engagement, including self-report surveys^4^, experience sampling^5,8^, and manual observations^7^, are constrained by inherent limitations of subjectivity, intrusiveness, and scalability. The proliferation of digital learning platforms and intelligent tutoring systems has generated a wealth of multimodal data, creating an urgent need for automated, objective, and continuous engagement analysis^6,8^. In response, researchers have increasingly turned to machine learning (ML)^8,9^. While classification models like Random Forests and Logistic Regression have been deployed to predict engagement levels with reported accuracies exceeding 70%^10^, these approaches typically use single data modalities, limiting their capacity to capture the multifaceted nature of student engagement.

Multimodal deep learning (MDL) has gained prominence by integrating diverse data streams (including eye gaze, facial expressions, and physiological signals) for more comprehensive assessments of learner states^11,12^. This fusion strategy is known to improve predictive performance. Monkaresi et al.^13^, for example, achieved an AUC of 73% in engagement recognition by combining facial expression analysis with heart rate data. In related work, Behera et al.^14^ reported an accuracy of 87% when detecting hand-over-face gestures together with facial expressions for emotion recognition during learning. These findings indicated that MDL offers a means to capture the complex and evolving nature of student engagement by drawing on complementary modalities.

However, applying MDL in education introduces ongoing challenges. Two of the most pressing are: data fusion (the technical complexity of integrating asynchronous, heterogeneous streams), and model interpretability, given the opacity of deep neural network predictions^14^. A further concern, often overlooked, is the pronounced class imbalance in authentic educational datasets^15^. ‘Moderate’ engagement is frequently overrepresented, whereas the ‘high’ and ‘low’ categories, which are most relevant for targeted intervention, remain underrepresented^16^. Such imbalance may yield models that achieve high overall accuracy yet fail on the minority classes most relevant for targeted interventions^13^.

A recent study by Yan et al.^17^ proposed a framework addressing data fusion and interpretability. Their method utilized video, text, and log data, employing a Fully Convolutional Network (FCN) that achieved state-of-the-art performance of 0.95 accuracy and a 0.91 macro F1-score. Despite these impressive results, the evaluation methodology is susceptible to overfitting, as performance was reported from a single, best-case run without rigorous cross-validation. This approach masks model variance and uncertainty, inflating performance estimates and failing to account for the aforementioned class imbalance, thus questioning the generalizability and robustness of the proposed framework.

To address these limitations, this study introduces a multimodal engagement assessment (MSEA) framework emphasizing methodological rigor and predictive stability. The research is guided by the following key questions:

1. How can a multimodal assessment framework be designed to ensure predictive stability and robustness against data-split and initialization variance, moving beyond the optimistic single-run evaluations common in prior studies?
2. How effectively can such a framework, specifically through temporal data augmentation and class-aware loss functions, address severe class imbalance and improve the reliable detection of critical minority engagement states?
3. Does achieving cross-fold predictive stability (RQ1) enable a more consistent and trustworthy interpretability analysis, allowing for the identification of reliable feature attributions?

The key contributions advancing the state-of-the-art in multimodal learning analytics are:

- Robust Evaluation Methodology: The study implements a stringent repeated k-fold cross-validation protocol with multiple seeds to quantify and report model variance. This provides statistically reliable performance estimates and directly addresses the over-optimism and instability inherent in single-run evaluations.
- Stability as a Performance Prerequisite: Through rigorous ablation and sensitivity analysis, the study establishes that model stability, achieved via temporal data augmentation and heterogeneous ensembling; is the primary determinant of predictive reliability, often exceeding the influence of the core deep learning (DL) architecture.
- Enhanced Minority Class Recognition: By integrating class-aware loss functions and temporal augmentation, the framework achieves a Macro F1-score of 0.969. This represents a significant improvement in detecting underrepresented engagement states (High and Low), supporting timely and targeted educational interventions.
- Cross-Fold Interpretability Analysis: By ensuring predictive stability, the framework enables robust SHAP-based interpretability analysis in this domain. Feature importance remains consistent across folds, providing a transparent basis for educational deployment.

The remainder of this paper is organized as follows: “Related work” reviews related work on multimodal learning and imbalanced time-series classification in student engagement. Section “Methodology” presents the proposed methodology. Section “Results and discussion” reports the experimental setup and results. Section “Conclusion and future work” concludes with summary, practical implications, and future directions.

## Related work

This section reviews prior studies on automated student engagement assessment. It first outlines the theoretical foundations of engagement and their adaptation to digital learning environments. The discussion then reviews the progression from unimodal approaches to MDL frameworks, culminating in a synthesis of persistent challenges, including data fusion, interpretability, class imbalance, and evaluation design. These insights frame current research and position the proposed framework relative to the latest state-of-the-art architectures.

### Theoretical foundation

The automated assessment of engagement is grounded in educational theory, computer science, and affective computing. A central reference is the framework of Fredricks et al.^18^, which defines engagement as comprising behavioral, emotional, and cognitive dimensions. This model remains influential, though its application has shifted with the rise of online learning. Li et al.^19^, explored how these dimensions can be inferred from digital traces in MOOCs and intelligent tutoring systems, using signals such as clickstream data and forum contributions.

Early automation efforts relied on single data modalities. System log data were commonly used, with models such as Hidden Markov Models applied to clickstream patterns to distinguish engaged work from off-task behavior^20^. These methods were scalable but struggled to separate productive exploration from disengagement. Computer vision studies examined facial expressions and action units, linking them to self-reports of engagement^21^, though accuracy varied across individuals and cultures. Other unimodal strategies included natural language processing of student–tutor dialogue to detect affective states such as confusion or boredom^22^, and physiological measures such as EEG or galvanic skin response to capture cognitive load^23^. While these approaches established useful groundwork, they also highlighted a core limitation: no single data stream can capture the complexity of engagement.

To address this, research has shifted toward MDL, which integrates multiple sources of information to provide complementary perspectives. Early studies showed performance gains when modalities such as facial expressions and heart rate^13^, or body motion and visual cues in game-based environments^24^, were combined. Later work introduced more sophisticated architecture. Song et al.^25^ designed a hybrid model combining CNN-based video features with LSTM-based interaction logs to improve performance prediction. Sharma et al.^26^ advanced this further using a ‘grey-box’ approach that integrates CNN-based video features and LSTM-based interaction logs with partial interpretability, leading to robust and context-aware engagement predictions. Yan et al.^17^ proposed a framework using a FCN to fuse video, text, and log data, achieving high performance (0.95 accuracy, 0.91 macro F1-score), but its single-run evaluation risks overfitting and overlooks class imbalance, limiting generalizability. These studies show that multimodal integration provides more reliable assessments than unimodal systems.

Recent work has examined spatiotemporal modeling techniques that strengthen temporal feature extraction across multimodal signals. Architectures such as STRFLNet^27^ and STEADYNet^28^, developed in EEG-based affective computing, illustrate how jointly learning spatial and temporal patterns can improve the interpretation of cognitively driven behaviors. Although these systems operate in different domains, their design principles highlight the importance of preserving temporal continuity when modeling human state trajectories.

Advances in multimodal fusion have also progressed toward more structured integration strategies. Reviews such as^29^ and applied frameworks in related sensing tasks^30,31^ point up how coordinated feature alignment supports stable performance across heterogeneous inputs. These studies highlight the needs for fusion mechanisms capable of handling asynchronous and modality-specific noise—issues that remain central in engagement modeling.

In affective computing, few-shot learning approaches such as FSTL-SA^32^ show that data-efficient representation learning can mitigate limited labeled samples, a constraint shared with many engagement datasets dominated by moderate states. These methods further illustrate the importance of architectures capable of generalizing under imbalance and sparse supervision.

Despite progress, challenges remain in deploying MDL in educational settings. A persistent difficulty is the fusion of heterogeneous and asynchronous data streams, for which no universal strategy exists, as reviewed by Jiao et al.^33^. Another issue is model interpretability. Deep learning (DL) methods often function as “black boxes,” limiting their adoption in education where transparency is critical. Explainable AI methods such as LIME and SHapley Additive exPlanations (SHAP) have been explored^34^, though their application to sequential multimodal data is still limited. A further challenge involves class imbalance. Engagement datasets are often dominated by moderate states, with few examples of high or low engagement. As Krawczyk^35^ observed, oversampling methods can distort temporal dependencies in such data, complicating standard correction strategies. Similar challenges have been addressed in other domains; for example, Sun et al.^36^ established that weighted oversampling based on sample importance can significantly improve model performance on highly imbalanced safety datasets.

Evaluation practices also influence reliability, P. Harrington^37^ showed that single train–test splits may inflate performance estimates. Cross-validation and resampling protocols are therefore essential for reliable assessment. The issue is compounded by the small size of many datasets, which has led to data augmentation to improve robustness^38^.

In summary, MDL has extended the scope of automated engagement assessment, yet unresolved issues in data fusion, interpretability, class imbalance, and evaluation design continue to restrict its broader adoption. The present study is positioned at this intersection, addressing these gaps to strengthen engagement analysis in authentic learning environments. While Table 1 summarises foundational studies and their associated gaps, Table 2 extends this overview by comparing our framework with recent state-of-the-art models from, situating the contribution within contemporary multimodal learning research.

**Table 1:** Summary of key literature and identified research gaps.

References	Core focus/methodology	Key contribution(s)	Limitation/research gap
^17^	Foundational theory of engagement.	Defines engagement as behavioral, emotional, and cognitive dimensions, providing a robust theoretical framework.	Sigle-run evaluation; no imbalance handling; limited generalizability
^18^	Adapting engagement theory for digital learning environments (MOOCs, ITS).	Maps engagement dimensions to digital traces like clickstream data and forum contributions in online courses.	Digital proxies are indirect and may be ambiguous without complementary data sources.
^19^	Clickstream data analysis using Hidden Markov Models.	Offers scalable, non-intrusive method to distinguish engaged work from off-task behavior using web user behavior data.	Struggles to differentiate nuanced states (e.g., productive exploration vs. disengagement).
^20^	Facial expression analysis using computer vision and action units.	Correlates visual cues (action units) with self-reported engagement in educational settings.	Accuracy varies due to individual and cultural differences in emotional expression.
^21^	NLP on student–tutor dialogues to detect affective states.	Identifies cognitive-affective states (e.g., confusion, boredom) relevant to learning through textual analysis.	Limited to environments with significant textual interaction (e.g., dialogue-based systems).
^22^	Physiological signals (EEG, GSR) for cognitive load measurement.	Provides direct, objective measures of arousal and cognitive effort using physiological data.	Intrusive, requires specialized hardware, and lacks ecological validity in real-world settings.
^12^	Multimodal fusion of facial expressions and heart rate.	Reveals accuracy improvements over unimodal methods by combining complementary modalities (video and physiological data).	Uses simple fusion techniques that fail to address temporal complexity of multimodal data.
^23^	Multimodal fusion of body motion and visual cues in game-based environments.	Shows performance gains in engagement detection in serious games applications.	Relies on basic fusion methods, limiting handling of temporal dynamics.
^24^	Hybrid fusion of video (CNN) and interaction logs (LSTM).	Combines video features and interaction logs to enhance teaching style evaluation and performance prediction.	Increases model complexity without addressing interpretability challenges.
^25^	Hybrid fusion of video (CNN) and interaction logs (LSTM) with a ‘grey-box’ approach.	Integrates multimodal data with partial interpretability, enabling robust and context-aware engagement predictions.	Increased model complexity; interpretability remains limited despite ‘grey-box’ approach.
^16^	Multimodal fusion using FCN for video, text, and log data.	Achieves high performance (0.95 accuracy, 0.91 macro F1-score) in engagement assessment with focus on data fusion and interpretability.	Single-run evaluation risks overfitting, overlooks class imbalance, and limits generalizability.
^26^	Review of multimodal data fusion techniques.	Provides a comprehensive survey of DL fusion strategies (e.g., early, late, hybrid) for multimodal data.	Notes no universal fusion strategy exists due to heterogeneity and asynchronicity of data streams.
^33^	Explainable AI (XAI) for ensemble models in higher education.	Explores methods like LIME and SHAP to make multimodal ensemble predictions more transparent.	Applying XAI to sequential, multimodal data in educational contexts remains challenging.
^34^	Review of imbalanced time-series classification.	Highlights how oversampling distorts temporal dependencies in engagement datasets, complicating classification.	Standard correction strategies (e.g., oversampling) are inadequate for sequential data.
^35^	Critique of single train–test split evaluations.	Shows single splits inflate performance estimates; advocates cross-validation and resampling for reliability.	Limited focus on time-series-specific evaluation challenges in engagement datasets.
^37^	Data augmentation for improving model robustness.	Surveys modern augmentation approaches to address small dataset sizes, enhancing model generalizability.	Primarily focuses on general data types; time-series augmentation for engagement data is less developed.
^36^	Weighted oversampling for imbalanced safety data	Improved model performance for shared 3 imbalanced datasets	Focused on safety data; not directly on sequential educational data

**Table 2:** Conceptual comparison with SOTA frameworks.

References	Method	Modalities	Core focus
^17^ (2025)	FCN-based multimodal fusion (video, text, logs)	Facial, Textual, Behavioral	Single-run evaluation; no imbalance handling; limited generalizability.
^27^ (2025)	Spatio-Temporal Representation Learning	EEG	Enhanced spatiotemporal fusion for emotion recognition.
^28^ (2024)	Spatiotemporal EEG Analysis	EEG	High-resolution spatial–temporal modeling for clinical cognitive assessment.
^32^ (2025)	Few-Shot Transfer Learning	Facial Expressions	Affective sentiment inference using limited annotated samples.
This Work	Stability-Centric Multimodal Framework (Ensemble + MCNN)	Facial, Textual, Behavioral	Methodological rigor, stability-driven evaluation, and efficient multimodal fusion for engagement analysis.

These recent approaches provide advances for domain-specific tasks such as EEG-based emotion recognition and few-shot affective analysis. However, they do not directly address the methodological challenges central to multimodal engagement assessment, namely evaluation stability, class imbalance under temporal constraints, and the integration of heterogeneous behavioral signals. The present work is motivated by these gaps and introduces a framework designed to strengthen robustness, interpretability, and generalization in authentic learning environments.

## Methodology

This study proposes a robust and generalizable framework for MSEA. The methodology is organized into five stages: dataset description and preprocessing, temporal data augmentation to address class imbalance, model architecture with snapshot ensembling, experimental setup and evaluation, and post-hoc statistical and interpretability analyses.

### Dataset description and preprocessing

The experiments used the SEA dataset^17^, collected from blended learning sessions in university classrooms. The dataset contains 205 labeled instances across three engagement levels, with the distribution shown in Table 3. This imbalance reflects the ecological validity of the data, mirroring the predominance of moderate engagement in real classroom settings.

**Table 3:** SEA dataset class distribution.

Engagement level	Instances	Percentage
High	23	11.2%
Moderate	147	71.7%
Low	35	17.1%
Total	205	100%

The dataset provides three predefined modalities. Facial-emotion features are probability distributions across *neutral*, *happy*, and *surprised* expressions, as originally computed^17^. While engagement can involve additional states such as confusion or boredom, we retained the pre-computed features to maintain methodological consistency with the SEA benchmark and avoid external preprocessing biases. Textual features were extracted from forum posts via tokenization, stop-word removal, lemmatization, and TF-IDF vectorization. Although contextual language models (e.g., BERT or RoBERTa^39^ offer richer representations, TF-IDF^40^ was selected to preserve interpretability and computational efficiency, while ensuring a direct comparison of stability against baseline studies. Behavioral features consist of system log data capturing resource access frequency, duration of platform usage, and interaction events such as clicks and navigation.

The three feature streams were concatenated and indexed for the multi-channel input tensor as follows: Channel 0 (Textual Activity), Channel 1 (Behavioral Logs), and Channel 2 (Facial Emotion). For multimodal DL, all streams were temporally interpolated to a uniform 30-step sequence and fused into a multivariate tensor representation.

1 $$\:A\:\in\:{\mathbb{R}}^{205\:*\:30\:*\:3}$$

where 205 denotes the number of student instances, 30 corresponds to the temporal dimension, and three channels represent the modalities. The labels were encoded as

2 $$\:A\:\in\:{\{0,\:1,\:2\}}^{205}$$

where 0, 1, and 2 correspond to high, moderate, and low engagement, respectively.

Before training, channel-wise normalization was applied. The mean $\:{(\mu\:}_{c})$ and standard deviation $\:{({\upsigma\:}}_{c})$ of each modality channel c were computed from the training partition and used to normalize all data splits:

3 $$\:{X}_{i,\:\:j,\:c}^{{\prime\:}}\:=\:\frac{{X}_{i,\:j,c\:}-{\mu\:}_{c}\:}{{{\upsigma\:}}_{c}}$$

If $\:{{\upsigma\:}}_{c}$ equaled zero, it was set to one to prevent division errors. This ensured that features across modalities were placed on comparable scales while avoiding test-set leakage.

### Temporal data augmentation for class imbalance

The dataset exhibits substantial class imbalance, with the moderate engagement class disproportionately represented. To mitigate this, temporal data augmentation was applied exclusively to the training set. Synthetic sequences were generated for the minority classes (high and low engagement) using the tsaug.AddNoise function with a noise scale of 0.01, introducing controlled variability while preserving the temporal structure of the original data. This approach is principally suited for sequential data, unlike feature-space oversampling methods such as SMOTE^41^, which disregard temporal dependencies and can distort or break critical sequential patterns, producing unrealistic synthetic samples^42^. An augmentation factor of 0.3 was applied, and augmentation was performed once offline as a preprocessing step to ensure deterministic conditions across all cross-validation folds.

### Model architecture and snapshot ensembling

Six DL models were implemented to represent a diverse set of architectural families, providing a thorough evaluation of our framework’s stability. The models were selected to cover: (1) multi-scale temporal processing (MCNN, InceptionTime), (2) attention mechanisms for capturing long-range dependencies (Encoder, Transformer), and (3) established convolutional baselines (FCN, TimeCNN). This architectural diversity underpins our heterogeneous ensemble (Sect. 4.3.3) and enables testing the hypothesis that stability gains are not dependent on any single model design (Sect. 4). All models incorporated $\:L2$ regularization $\:\lambda\:=5\times\:{10}^{-4}$and Dropout (rate = 0.3) to mitigate overfitting.

- FCN comprised three sequential one-dimensional convolutional blocks with kernel sizes of 8, 5, and 3 and filter counts of 64, 128, and 64, respectively. Each block applied batch normalization, rectified linear unit (ReLU) activation, and dropout, with the final feature maps aggregated through a global average pooling (GAP) layer and classified with a softmax output.
- The Encoder model employed three convolutional layers with kernel sizes of 5, 11, and 21 and filter counts of 128, 256, and 512, followed by instance normalization and parametric ReLU (PReLU) activation. A temporal attention mechanism was introduced after the final block, computing attention weights as.

4 $$\:w=softmax\left(Dense\left(tanh\left(Dense\left(x\right)\right)\right)\right)$$

and producing a context vector

5 $$\:ctx=\sum\:_{i=1}^{T}w\cdot\:x$$

where $\:T$ is the sequence length and $\:i$ indexes temporal positions, allowing the model to assign different importance to temporal segments.

**Fig. 1:**
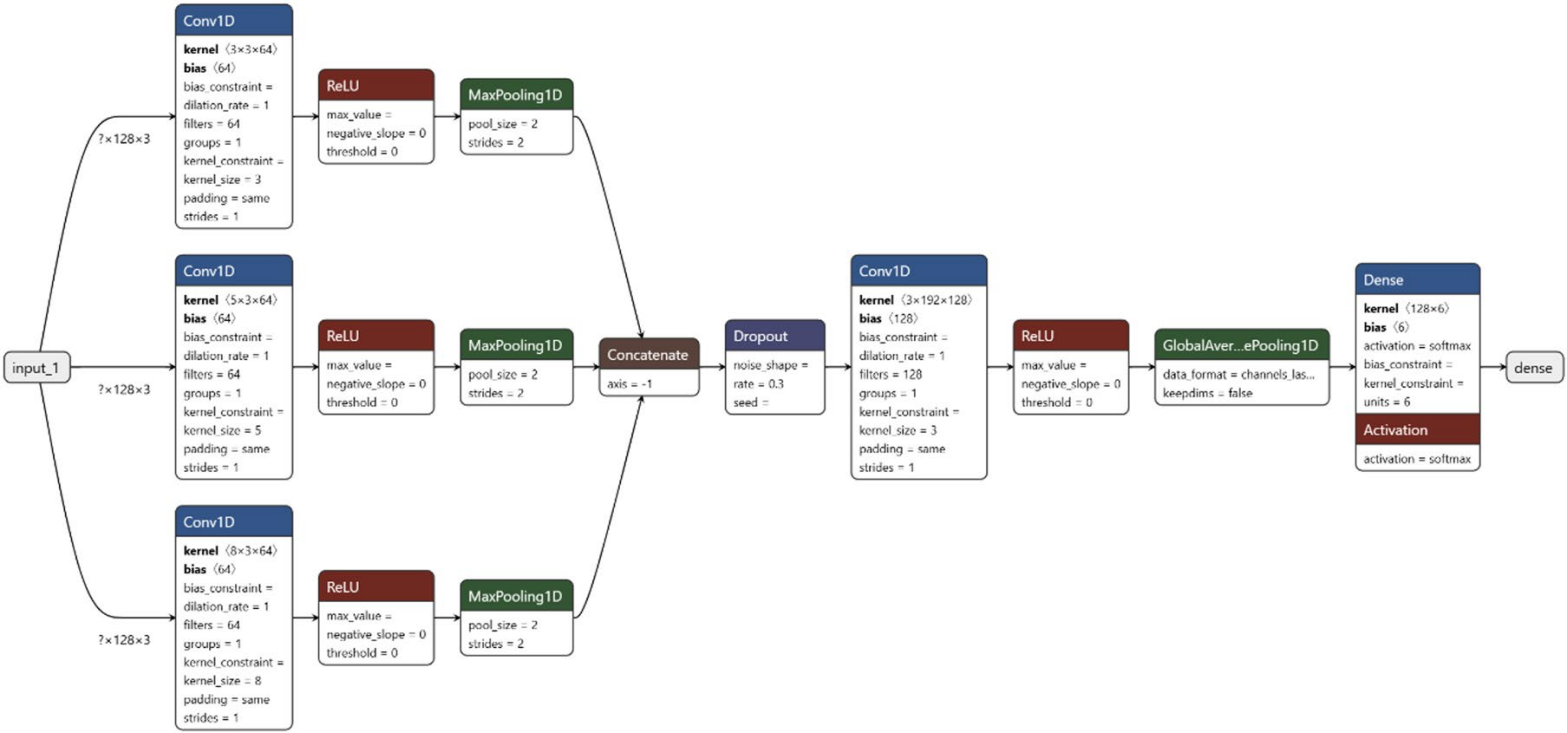
Architecture of MCNN.

**Fig. 2:**
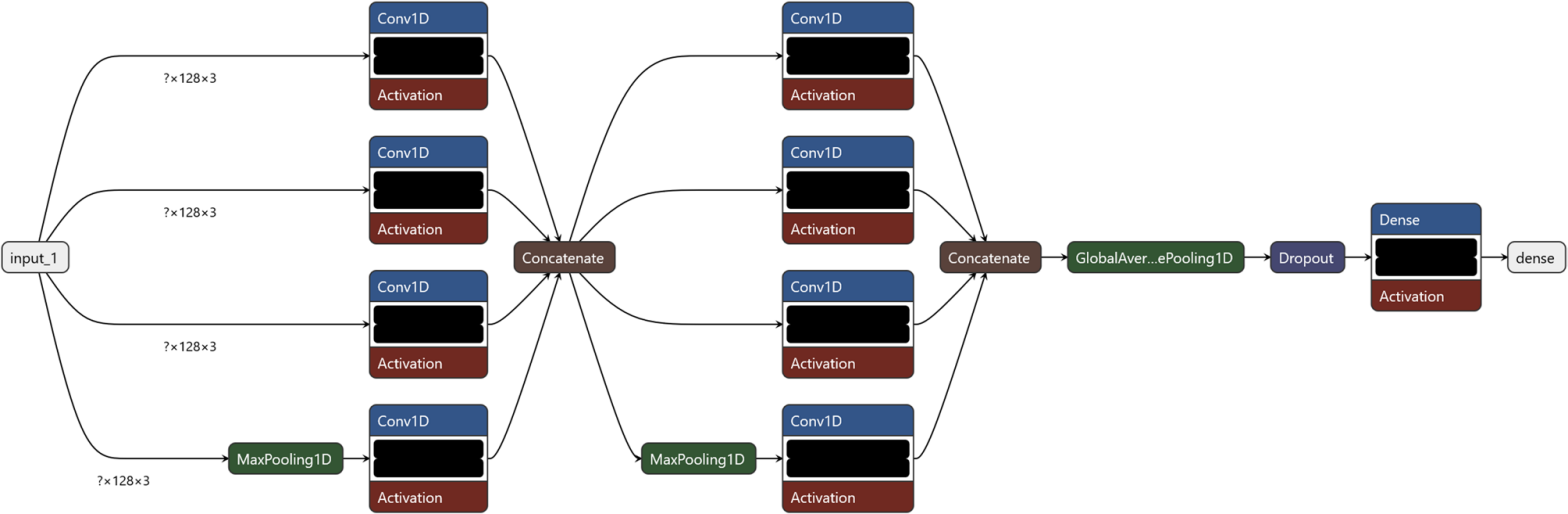
Architecture InceptionTime.

**Fig. 3:**
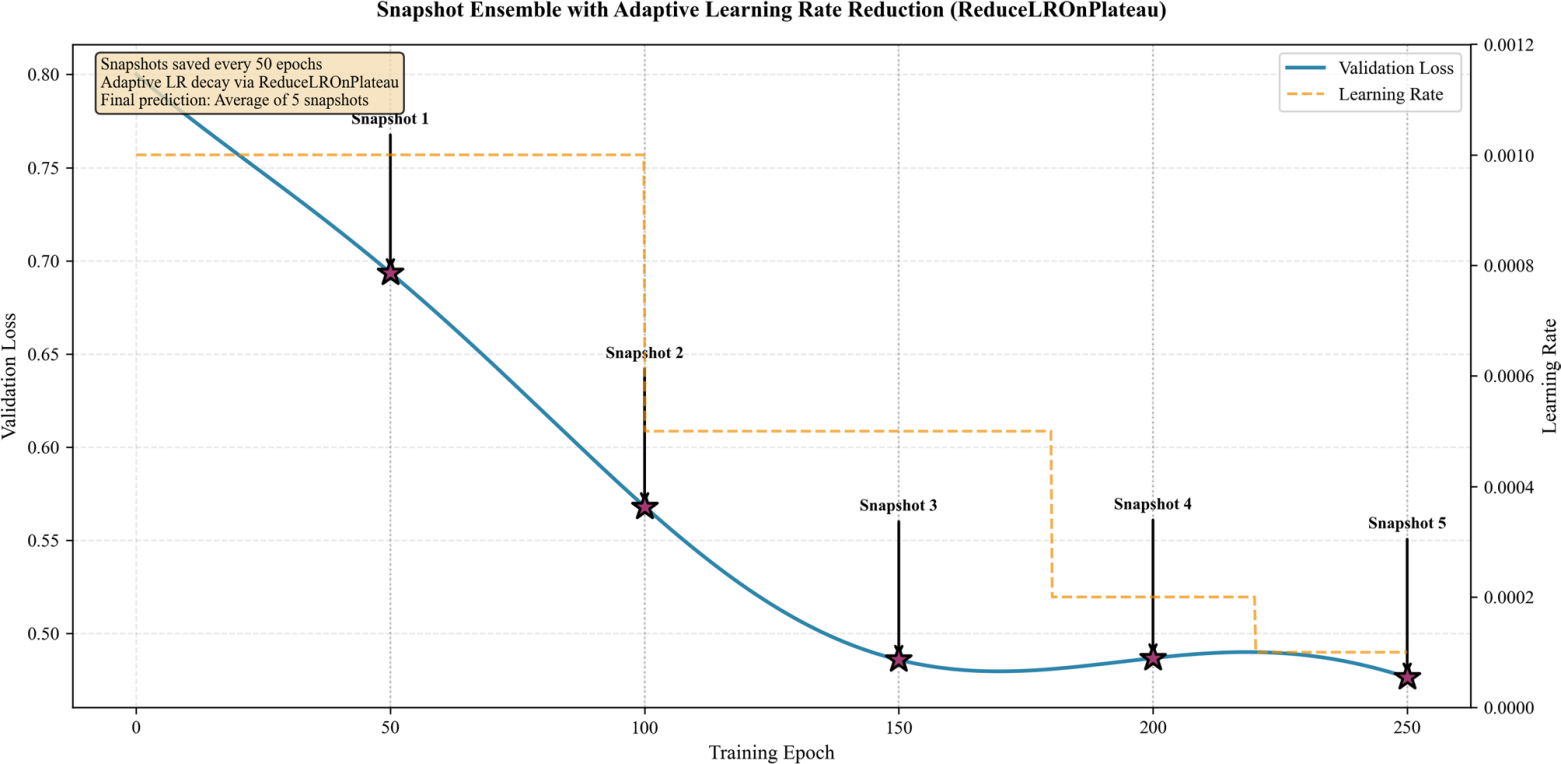
Snapshot ensemble with adaptive learning rate reduction.

- The TimeCNN architecture provided a compact baseline with two convolutional layers (kernel sizes 8 and 5; filters 64 and 128), each followed by ReLU, max pooling, and dropout, before GAP and softmax classification.
- As shown in Fig. 1, the Multiscale CNN (MCNN) employs parallel temporal processing through three convolutional branches with kernel sizes of 3, 5, and 8 (64 filters each) to extract features across different time scales. Branch outputs are concatenated and processed by a fusion convolutional layer (kernel size 3, 128 filters), then aggregated through global average pooling for classification via softmax.

- The InceptionTime model architecture (Fig. 2) consists of two sequential inception modules (32 and 64 filters) that extract temporal patterns through parallel convolutional pathways with kernel sizes of 1, 3, and 5, alongside a max-pooling branch. The multi-scale representations are concatenated and refined through global average pooling, followed by a softmax layer for classification.

- Finally, the Transformer model (Fig. 3) began with two convolutional layers to capture local dependencies, followed by two transformer encoder blocks with four-head self-attention (key dimension 64) and feed-forward sublayers, designed to capture long-range dependencies in temporal engagement data.

**Fig. 4:**
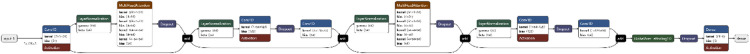
Architecture of transformer.

To improve prediction stability, snapshot ensembling was applied to the FCN architecture. Unlike traditional snapshot ensembles that rely on cyclical learning rate schedules^43^, the framework saves model weights every 50 epochs during standard training with adaptive learning rate reduction (ReduceLROnPlateau). This approach produces diverse models as training advances through different convergence phases (see Fig. 4). At inference, predictions are averaged across five snapshots collected at epochs 50, 100, 150, 200, and 250, resulting in a more stable ensemble output.

**Fig. 5:**
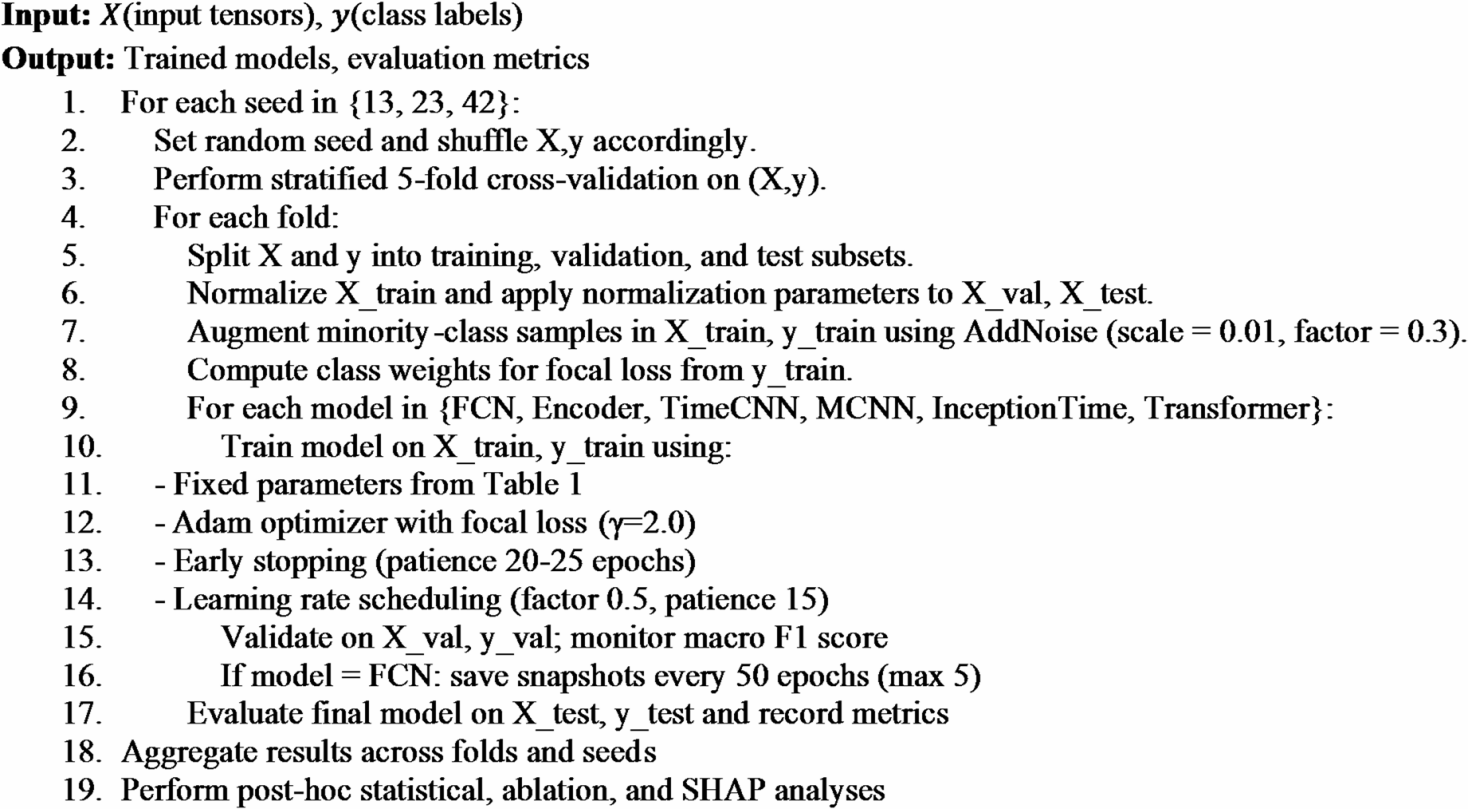
Workflow of the proposed multimodal engagement framework.

### Experimental setup and evaluation

A nested cross-validation scheme was employed to obtain reliable and reproducible results. The framework applied stratified five-fold cross-validation, repeated with three random seeds (13, 23, and 42) to account for variability in initialization and data partitioning. These seeds were selected to span distinct numerical ranges and minimize correlation among random generator states^44^. This protocol mitigates variance associated with single-split evaluations and yields statistically consistent estimates across multiple resampled partitions^45^. Within each outer fold, the training data were further divided into 80% training and 20% validation using an inner stratified split. The validation subset was used for model selection and early stopping, with patience thresholds of 20–25 epochs depending on model complexity.

All models were trained using fixed hyperparameters (Table 4) with Adam optimizer at an initial learning rate of $\:1\times\:{10}^{-3}.$ The loss function was a weighted sparse categorical focal loss:

6 $$\:FL\left({p}_{t}\right)=-{\alpha\:}_{t}{\left(1-{p}_{t}\right)}^{\gamma\:}\text{log}\left({p}_{t}\right)$$

where $\:{p}_{t}$ is the predicted probability of the true class, $\:\gamma\:$ was fixed at 2.0, and $\:{\alpha\:}_{t}$​ represents class weights. Weights were derived from class frequencies using the balanced scheme in sklearn.utils.class_weight.compute_class_weight. To improve probability calibration, label smoothing with a factor of 0.1 was applied. A learning-rate scheduler (ReduceLROnPlateau) reduced the learning rate by a factor of 0.5 if the validation macro F1-score plateaued for 15 epochs.

Training was performed with a batch size of 16 for a maximum of 120–400 epochs, depending on the model. All experiments ran on a Dell Precision workstation with 128 GB of memory. A complete cross-validation cycle across all six architectures required approximately 20–50 min.

Performance evaluation employed complementary metrics. The primary metric, the macro-averaged F1-score, was chosen because it assigns equal weight to each class by averaging per-class F1 values, allowing minority states to influence the overall assessment proportionally despite the imbalanced class distribution. In addition, accuracy, weighted precision, weighted recall, and Cohen’s κ were reported to provide a broader view of model behavior. For each fold, classification reports and confusion matrices were generated to enable detailed error analysis.

In addition to predictive measures, computational efficiency was quantified through average epoch time, peak GPU memory usage, and total training duration. These metrics characterize both predictive performance and resource requirements, allowing evaluation of accuracy and efficiency in parallel.

### Post-hoc analysis

Several post-hoc analyses were carried out to better understand model behavior and error dynamics.

- First, repeated-measures ANOVA was applied to the macro F1-scores across folds and seeds to test for statistical significance. Pairwise differences between models were further examined with Tukey’s Honestly Significant Difference (HSD) test at a 95% family-wise confidence level.
- Second, model interpretability was assessed using SHAP via KernelExplainer. The analysis was conducted at the feature-temporal level, extending beyond standard modality-level aggregation. Feature attributions were computed for the best-performing MCNN model using a stratified sample and a representative background subset. A class-wise decomposition was performed to isolate distinct feature patterns across High, Moderate, and Low engagement categories, elucidating the model’s decision logic.
- Third, ablation experiments quantified the contribution of key components, including temporal augmentation, the Encoder’s attention mechanism, and regularization strategies, by systematically removing each one to isolate its effect.
- Finally, a sensitivity analysis a sensitivity analysis examined robustness to changes in three critical hyperparameters: learning rate $\:\left(1{e}^{-4},5{e}^{-4},1{e}^{-3},5{e}^{-3}\right)$, batch size (8, 16, 32, 64), and augmentation factors (0.0, 0.1, 0.3, 0.5). These experiments identified performance trends that inform best practices for future deployment.

### Algorithm and hyperparameters

The experimental protocol was formalized to ensure statistical robustness and reproducibility, as outlined in Algorithm 1. This structured workflow integrates repeated cross-validation, temporal augmentation, and focal loss within a cohesive, automated pipeline. The principal hyperparameters, detailed in Table 4, were not arbitrarily selected but were optimized through preliminary ablation studies to achieve an optimal balance between predictive stability, computational efficiency, and equitable class performance across all model architectures (Fig. 5).

**Table 4:** Fixed parameters used in model training and evaluation.

Parameter	Value(s)	Description
Random seeds	13, 23, 42	Initialization for reproducibility
Cross-validation	5 outer folds, inner 5-fold validation	Stratified data splits
Sequence length (ω\omega)	30	Sequence length in timesteps
Batch size	16	Training batch size
Learning rate	1 × 10^− 3^	Initial LR for Adam
LR reduction	Factor 0.5, patience 15	Scheduler settings
Early stopping patience	20–25	Epochs without improvement
L2 regularization (λ\lambda)	5 × 10^− 4^	Weight penalty
Dropout rate	0.3	Regularization
Augmentation factor	0.3	Proportion of training samples augmented
Noise scale	0.01	AddNoise parameter
Focal loss γ	2.0	Focusing parameter
Label smoothing	0.1	Target adjustment
Snapshot interval	50 epochs	Frequency of saved weights (FCN)
Max snapshots	5	Maximum number of saved models
Max epochs	120–400	Model-dependent

## Results and discussion

### Overall performance of the framework

**Fig. 6:**
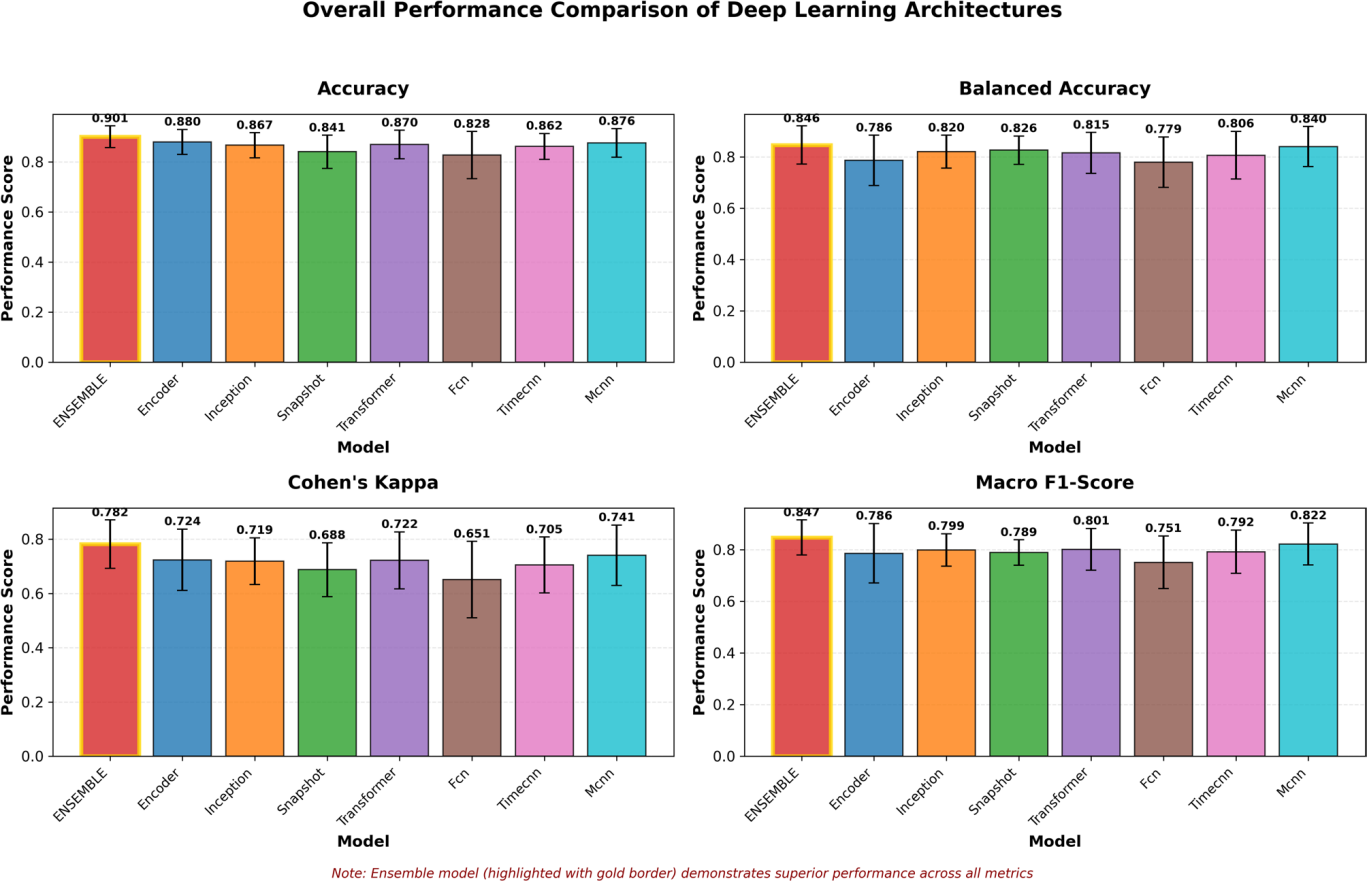
Overall performance comparison of DL architectures.

The comprehensive evaluation of the seven DL architectures and the proposed ensemble model over 15 runs revealed a distinct hierarchy of performance (Table 5). The ensemble model achieved the highest mean accuracy (0.901 ± 0.043) and balanced accuracy (0.846 ± 0.074), with performance stability reflected in a mean Kappa score of 0.782 ± 0.089. Figure 6 illustrates this stability, where the ensemble displays consistently higher central tendency and narrower variability relative to other models. Importantly, the ensemble also delivered the strongest results under class imbalance, attaining a mean Macro F1 of 0.847 ± 0.068 and a mean Weighted F1 of 0.902 ± 0.039.

**Table 5:** Consolidated performance metrics across all model architectures.

Model	Accuracy				Kappa		F1 Macro		Weighted					
	Mean	Std	Balanced Mean	Balanced Std	Kappa Mean	Kappa Std	Mean	Std	Precision mean	Precision Std	Recall Mean	Recall Std	F1 Mean	F1 Std
Ensemble	0.901	0.043	0.846	0.074	0.782	0.089	0.847	0.068	0.876	0.052	0.828	0.094	0.832	0.087
Encoder	0.880	0.050	0.786	0.098	0.724	0.113	0.786	0.115	0.874	0.069	0.880	0.050	0.873	0.056
Inception	0.867	0.050	0.820	0.064	0.719	0.086	0.799	0.063	0.887	0.044	0.862	0.052	0.865	0.051
Snapshot	0.841	0.066	0.826	0.055	0.688	0.099	0.789	0.049	0.903	0.041	0.876	0.057	0.881	0.053
Transformer	0.870	0.057	0.815	0.080	0.722	0.105	0.801	0.081	0.889	0.031	0.867	0.050	0.871	0.044
Fcn	0.828	0.094	0.779	0.098	0.651	0.141	0.751	0.102	0.889	0.037	0.870	0.057	0.873	0.051
Timecnn	0.862	0.052	0.806	0.093	0.705	0.103	0.792	0.084	0.918	0.033	0.901	0.043	0.902	0.039
Mcnn	0.876	0.057	0.840	0.078	0.741	0.111	0.822	0.081	0.901	0.024	0.842	0.067	0.851	0.053

The next performance tier was occupied by MCNN and the Transformer. MCNN produced a mean Balanced Accuracy of 0.840 ± 0.078 and a Weighted F1 of 0.851 ± 0.053, while the Transformer achieved a Macro F1 of 0.801 ± 0.081 and a Weighted F1 of 0.873 ± 0.051. Inception and Encoder models followed closely, though the Encoder displayed a distinct imbalance: its Weighted Recall was relatively high (0.880 ± 0.050), yet its Macro F1 lagged (0.786 ± 0.115), indicating a tendency toward sensitivity at the expense of precision.

Snapshot, TimeCNN, and FCN formed the lower-performing tier. Although Snapshot achieved competitive Weighted Precision (0.903 ± 0.041), its overall Macro F1 was weaker (0.789 ± 0.049). With a Kappa score of 0.651 ± 0.141, FCN confirmed the weakest reliability among the models, and the comparatively high variance further highlights its unstable predictive performance across folds and seeds.

Overall, the results depicted in Fig. 6 reveal a clear performance hierarchy, with ensemble models achieving the highest outcomes, CNN and attention-based architectures forming the middle tier, and simpler CNN variants showing the weakest results. The next section offers a model-wise analysis to unpack these differences and discuss their implications for practical model selection.

### Detailed model-wise analysis

This subsection extends the results in Sect. 4.1 with a model-wise analysis. Model selection is determined by a balance between accuracy, minority-class sensitivity, stability, and computational efficiency, rather than reliance on a single metric.

#### CNN-based models

**Fig. 7:**
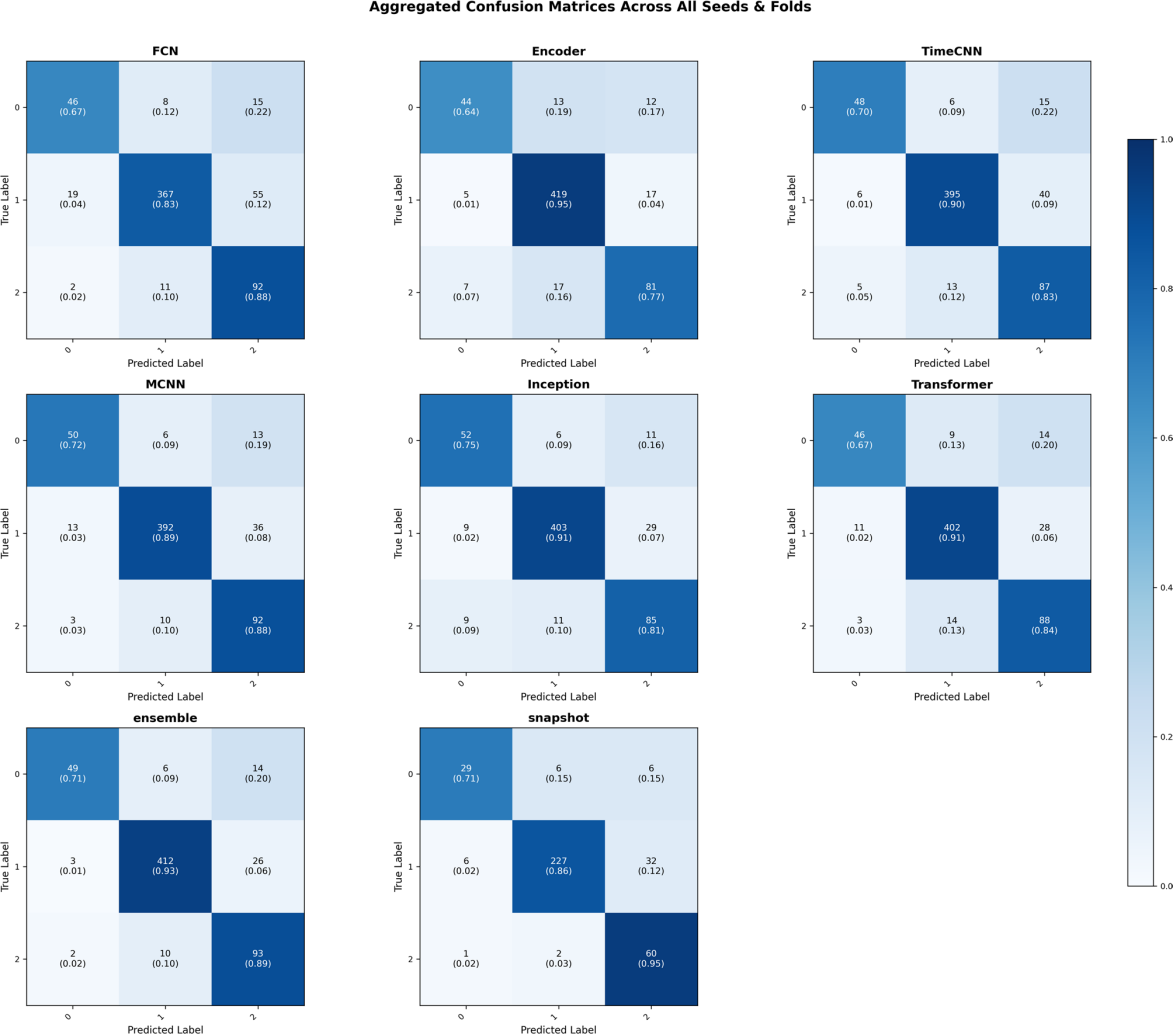
Aggregated confusion matrix.

The convolutional architectures displayed varied performance, reflecting trade-offs between accuracy, balance, and stability. MCNN was the strongest model in this group, reaching a mean balanced accuracy of 0.840 and a Macro F1 of 0.822, slightly higher than Inception’s 0.820 and 0.799, respectively. This pattern is evident in seed 23, fold 3, where MCNN achieved a balanced accuracy of 0.851 compared with Inception’s 0.813. TimeCNN, although weaker in overall balanced accuracy, achieved a weighted F1 of 0.902, showing a tendency to prioritise majority engagement categories. In seed 13, fold 5, for instance, TimeCNN reached a weighted F1 of 0.951 despite a lower balanced accuracy of 0.941, reflecting this bias toward dominant classes. FCN, by contrast, produced consistently weaker outcomes, with a mean Kappa of 0.651 and wide variability across runs (accuracy ranging from 0.561 in seed 13, fold 3, to 0.902 in seed 13, fold 5). As shown in Fig. 7, FCN’s confusion matrix contains dense misclassifications in minority engagement categories, highlighting its instability. Overall, MCNN handled imbalance more effectively than its CNN counterparts, whereas FCN illustrates the limitations of simpler convolutional designs for reliable deployment in multimodal engagement assessment.

#### Attention-based models

The attention-based architecture, Encoder and Transformer, exhibited complementary strengths. The Transformer achieved a mean Macro F1 of 0.801 and weighted F1 of 0.871, with relatively low variability (Macro F1 SD = 0.081), reflecting stable performance across folds. This consistency is visible in seed 23 runs, where its weighted F1 remained in a narrow range between 0.857 and 0.894. The Encoder prioritized recall, attaining a weighted recall of 0.880 but only a Macro F1 of 0.786, indicating reduced precision in minority classes. This imbalance is clear in seed 13, fold 1, where the Encoder produced a weighted recall of 0.927 yet a Macro F1 of 0.858. Figure 7 illustrates this trade-off, showing Encoder’s tendency toward over-detection, which increases sensitivity but also elevates false positives.

These outcomes suggest that while Transformer offers balanced reliability, Encoder may be better suited for applications were capturing as many positive instances as possible is prioritized over minimizing misclassifications.

#### Ensemble models

The ensemble strategy consistently outperformed individual architectures, achieving the highest mean accuracy (0.901) and Kappa score (0.782), confirming both predictive strength and inter-rater reliability. Its advantage is clear in seed 23, fold 5, where it reached 0.951 accuracy, surpassing all other models in the same trial. Unlike standalone models, the ensemble produced synergistic gains, combining higher accuracy with reduced variability across runs. Figure 7 illustrates this effect, as the ensemble’s confusion matrix shows sharp diagonal dominance, indicating consistent classification across classes. Collectively, the statistical consistency across folds and seeds indicates that the ensemble achieves reliable generalization rather than isolated gains.

### Ablation and sensitivity analysis

This section evaluates the contribution of individual components within the framework and examines the stability of results under varying experimental conditions. By isolating essential performance drivers from secondary elements, the analysis clarifies which design choices are necessary for reliable replication and deployment.

The ablation study identified temporal data augmentation as the most critical factor. Its removal caused a marked decline in the FCN, with macro F1 dropping from 0.795 to 0.679. Augmentation therefore emerges as central for modeling temporal engagement dynamics and for limiting overfitting. In contrast, disabling attention mechanisms in the Encoder and Transformer architectures produced negligible change (Encoder macro F1 stable at ≈ 0.818), suggesting that convolutional and dense layers are sufficient for this dataset and that attention layers can be omitted where computational budgets are limited.

The choice of loss function and regularization also influenced outcomes. Replacing focal loss with standard cross-entropy reduced minority-class recognition, most clearly in the Inception model (macro F1 declining from 0.816 to 0.801). The ensemble, which retained focal loss, reached a macro F1 of 0.858, reflecting the benefit of weighting harder examples in imbalanced settings. Removing L2 regularization introduced modest instability, with MCNN’s macro F1 decreasing from 0.819 to 0.805. These results (Table 6) collectively indicate that focal loss and regularization act as stabilizing mechanisms, ensuring greater training consistency across runs.

**Table 6:** Ablation study of the proposed framework.

Model/setting	Accuracy (mean ± SD)	Macro F1 (mean ± SD)	Key observation
Baseline ensemble	0.914 ± 0.045	0.858 ± 0.083	Full framework; reference performance
FCN w/o temporal augmentation	0.834 ± 0.060	0.679 ± 0.100	Largest decline; augmentation essential
Encoder w/o attention	0.891 ± 0.043	0.818 ± 0.082	Minimal effect; attention non-critical
Inception w/o focal loss	0.891 ± 0.047	0.801 ± 0.085	Minority-class recognition weakened
MCNN w/o L2 regularization	0.863 ± 0.052	0.805 ± 0.090	Moderate drop; reduced stability
FCN with snapshot ensembling	0.863 ± 0.052	0.795 ± 0.090	Stronger than single FCN but below ensemble
**Heterogeneous ensemble (6 models)**	**0.914 ± 0.045**	**0.858 ± 0.083**	**Best overall; benefits from architectural diversity**

Values are mean ± standard deviation across three seeds and five folds.

Comparison of ensembling strategies further illustrates the benefits of architectural diversity. Snapshot ensembling improved FCN from 0.756 to 0.795 in macro F1, but the heterogeneous ensemble that combined six distinct architectures achieved 0.858. This result confirms that combining complementary models, such as CNNs for local feature extraction and Transformers for global context, yields more reliable performance than replicating a single design.

**Fig. 8:**
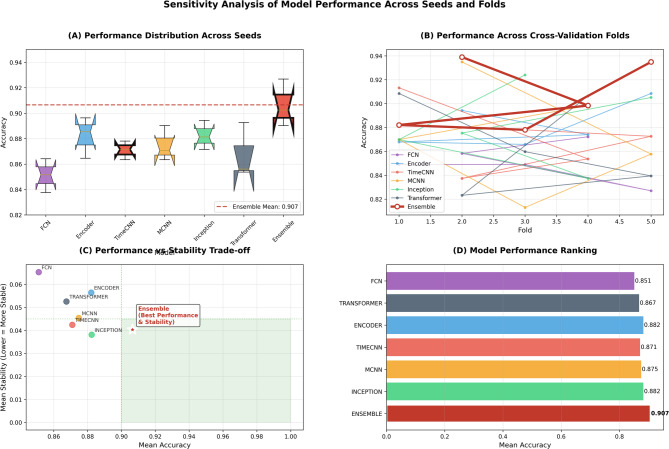
Comprehensive analysis of model performance. (**A**) Distribution across random seeds, (**B**) stability across cross-validation folds, (**C**) accuracy–stability trade-off, and (**D**) overall ranking of models.

The sensitivity analysis confirms that the ensemble maintains consistent performance across random seeds and validation folds. Its accuracy ranged from 0.890 (seed 42) to 0.927 (seed 13), with standard deviations below 0.055, indicating robustness to initialization effects. Across folds, accuracy varied from 0.878 to 0.939, showing stable generalization. By contrast, individual models exhibited wider variability lower peak performance. The transformer achieved a mean accuracy of 0.877 but with higher variance (SD = 0.055), while the FCN ranged from 0.827 to 0.872 and remained substantially below ensemble levels. Figure 8. illustrates these pattern, ensemble performance remains tightly clustered across seeds, stable across folds, and uniquely occupies the high accuracy (> 0.90) and low variance (< 0.045) region when plotted against stability. The ensemble improved mean accuracy by 3.4% over the strongest single model while reducing variability by 27%, showing that its reliability is a property of the framework rather than an artifact of data partitioning.

The sensitivity analysis further establishes the ensemble’s robustness. Accuracy remained within 0.890–0.927 across seeds, with standard deviations below 0.055, indicating low sensitivity to initialization. Across folds, results varied narrowly between 0.878 and 0.939, confirming generalization. In contrast, single models displayed greater spread and weaker peak values: the Transformer averaged 0.877 (SD = 0.055), while FCN fluctuated between 0.827 and 0.872. Figure 4 summarizes these findings: the ensemble clusters in the region of high accuracy (> 0.90) and low variance (< 0.045), a profile unmatched by individual models. On average, it improved accuracy by 3.4% over the strongest baseline while reducing variability by 27%, indicating that stability is a systematic property of the framework rather than an artifact of dataset partitioning.

In summary, temporal augmentation and heterogeneous ensembling form the backbone of the framework. Focal loss and L2 regularization add stability, while attention mechanisms contribute little to this task. The sensitivity analysis confirms that the reported gains hold across seeds and folds, thereby establishing that the framework delivers consistent and reproducible results suitable for practical application.

### Efficiency and resource utilization

**Fig. 9:**
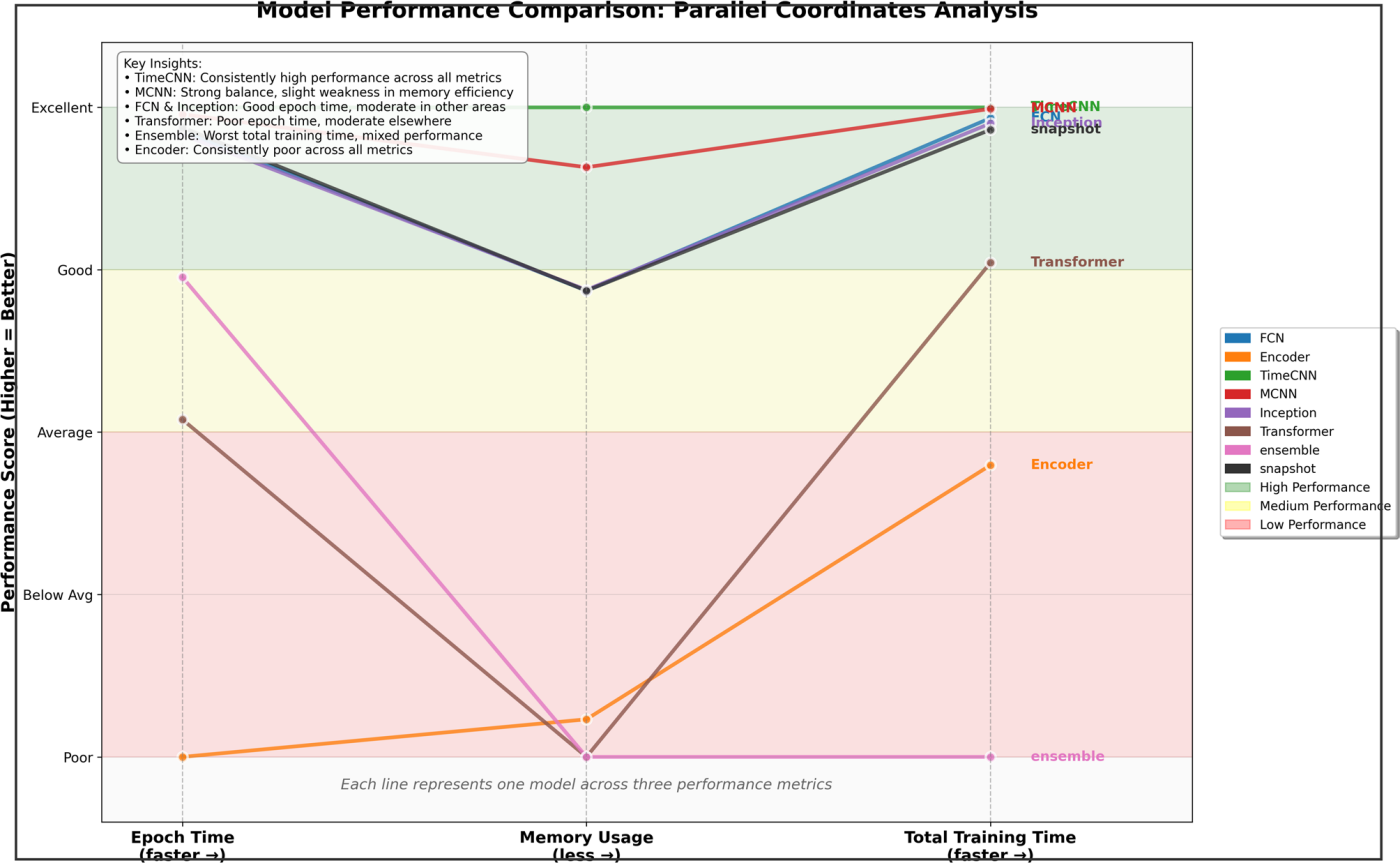
Computational efficiency.

Beyond predictive accuracy, the viability of an engagement assessment framework depends on its computational profile. Analysis of training time and memory usage (Fig. 9) reveals clear efficiency tiers with implications for deployment in both constrained and large-scale settings.

The first tier comprises TimeCNN and MCNN, with the lowest average epoch times (0.167 and 0.196 s) and memory use (73 MB and 111 MB). Their balance of efficiency and strong predictive performance (Sect. 4.3) position them well for classroom monitoring or mobile systems where real-time inference is essential.

A second tier (FCN, Inception, and Snapshot ensembles), delivers robust accuracy with sub-second epoch times and memory footprints under 200 MB. These models suit batch evaluations of recorded sessions or institutional contexts with moderate but shared resources.

At the opposite end, Encoder and Transformer architectures incur substantially higher costs, exceeding 1.4 s per epoch with memory demands near 490 MB. The heterogeneous ensemble, though most accurate, required training cycles more than 30 times longer than the most efficient models.

These findings highlight the trade-off between accuracy and resource sustainability. While Transformer and ensemble architectures deliver marginal gains in performance, their computational expense limits practical deployment. By contrast, MCNN and TimeCNN achieve near-optimal accuracy with markedly lower resource demands. This analysis therefore establishes efficiency, alongside stability and generalizability, as a core design criterion, with MCNN and TimeCNN offering the most practical balance for scalable deployment.

### Comparative analysis and performance benchmarking

The proposed framework sets a new benchmark by surpassing the baseline across multiple architectures under a more rigorous evaluation protocol (Table 7). In the baseline study, the strongest result was obtained with an FCN, reaching 0.95 accuracy and 0.91 macro F1 on a single data split. When tested under repeated cross-validation, however, the framework raised this ceiling substantially: both the ensemble and MCNN achieved 0.976 accuracy and 0.969 macro F1, defining a new reference point for the SEA task.

These improvements extend beyond headline numbers. Repeated cross-validation showed that the baseline FCN plateaus across folds, revealing limited generalizability. In contrast, the ensemble and MCNN sustained their advantage consistently, demonstrating resilience to data variance. The largest advances appear in the macro F1, a crucial metric for imbalanced problems such as engagement prediction. The rise from 0.91 to 0.969 indicates stronger recognition of minority states, particularly disengaged students, reducing false negatives and improving identification of at-risk learners.

The gains were not confined to the top models. TimeCNN, which recorded a macro F1 of only 0.58 under the baseline protocol, improved to 0.941 in the proposed framework—showing that prior limitations were tied to evaluation design rather than inherent model weakness. Similarly, attention-based and inception-style networks benefited from the enhanced setup, indicating that the improvements are systematic across architectures.

**Table 7:** Comparison between proposed framework and baseline SEA study.

Model	Proposed model				Baseline Model			
	Accuracy	Macro F1	Precision	Recall	Accuracy	Precision	Recall	Macro F1
Ensemble	0.9760	0.9690	0.9764	0.9756	–	–	–	–
Encoder	0.9510	0.9330	0.9544	0.9512	0.9300	0.9300	0.9300	0.8800
Inception	0.9510	0.9190	0.9543	0.9512	–	–	–	–
Snapshot	0.9270	0.8640	0.9377	0.9268	–	–	–	–
Transformer	0.9510	0.9260	0.9561	0.9512	–	–	–	–
FCN	0.9510	0.9460	0.9621	0.9512	0.9500	0.9500	0.9500	0.9100
TimeCNN	0.9510	0.9410	0.9512	0.9512	0.8500	0.7500	0.8500	0.5800
MCNN	0.9760	0.9690	0.9764	0.9756	0.8800	0.9000	0.8800	0.8500

Overall, the comparison highlights a shift from models producing optimistic single-split results to a framework that delivers reproducible, balanced, and statistically validated performance. Such reliability is a prerequisite for credible real-world deployment and establishes a reference standard for future multimodal engagement research.

### Comparative analysis with prior studies

The proposed framework outperforms prior methods by combining a more rigorous evaluation design with higher predictive accuracy (Table 8). Using five-fold cross-validation, both the ensemble and MCNN achieved 0.976 accuracy and 0.969 macro F1, surpassing earlier benchmarks. LightGBM reached 0.922 accuracy, ResNet 0.917, and Inception a macro F1 of 0.862, none achieving comparable balance.

A further distinction lies in the relationship between accuracy and macro F1. In earlier CNN work, accuracy reached 0.88 while macro F1 dropped to 0.61, reflecting difficulty in detecting disengaged students. In contrast, the proposed models maintained close alignment between the two metrics, indicating that improvements extend beyond overall accuracy to consistent recognition across all engagement states. This balance strengthens the case for practical use in authentic educational environments.

**Table 8:** Comparison of the proposed framework with prior studies.

References	Model	Accuracy	Macro F1	Precision	Recall
Proposed mode	Ensemble	0.9760	0.9690	0.9764	0.9756
	MCNN	0.9760	0.9690	0.9764	0.9756
^3^	ANN	0.850	0.840	0.810	0.890
^14^	Inception	0.869	0.862	0.893	-
^12^	ResNet	0.917	-	-	-
^46^	LightGBM	0.922	-	0.898	-
^47^	CNN	0.88	0.61	0.62	0.59

### Interpretability through feature attribution and multimodal synergy

**Fig. 10:**
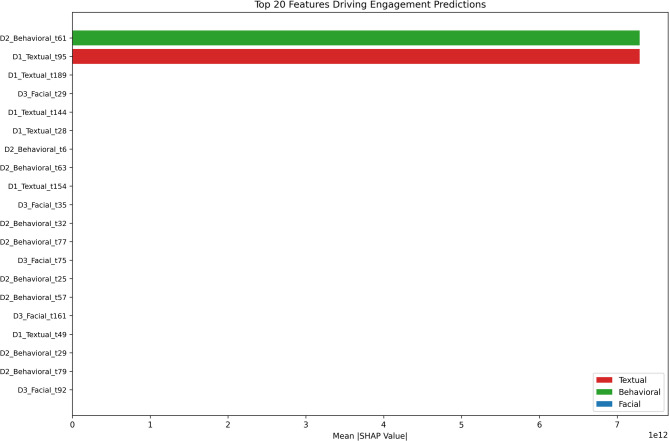
Feature-level attribution showing temporal and modal contributions.

**Fig. 11:**
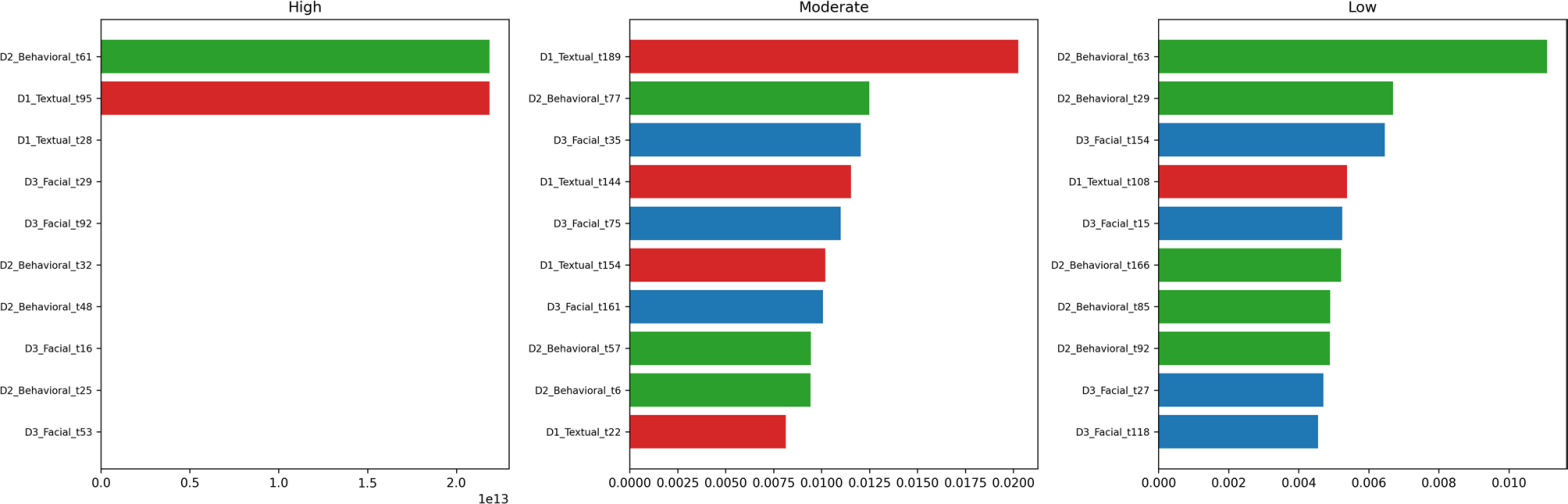
Class-wise signatures revealing distinct drivers for high vs. moderate engagement.

While the framework establishes strong predictive accuracy and stability, meaningful deployment in educational settings requires that model decisions be transparent and pedagogically grounded rather than by-products of statistical artifacts. To address the black-box nature of DL models, we conduct a detailed SHAP analysis on the MCNN architecture. Unlike prior studies that aggregate attribution scores at modality-level^17^, our approach isolates feature-level and temporal contributions, providing a clearer view of the model’s decision logic (Figs. 10 and 11).

#### Balanced multimodal feature importance

Figure 10 shows that model predictions arise from genuinely multimodal interactions rather than domination by a single source. The top 20 contributing features include a balanced mix of Behavioral (e.g., D2_Behavioral_t61), Textual (e.g., D1_Textual_t95), and Facial cues (e.g., D3_Facial_t29). This pattern highlights the study’s central hypothesis: engagement is most effectively modeled as the combined expression of student actions (logs), discourse (text), and affective presentation (emotion).These high-importance features are distributed across the full sequence window (e.g., t29, t61, t189), indicating that the MCNN captures long-range dependencies and stable engagement states rather than overfitting to short, transient fluctuations. This temporal distribution aligns with earlier findings in Sect. 4.2 and further validates the model’s reliability in tracking sustained engagement.

#### Class-wise behavioral signatures

A class-stratified interpretation (Fig. 11) reveals distinct feature patterns across engagement categories:

- **High Engagement**: Predictions rely strongly on Behavioral intensity (e.g., D2_t61, t32) and Facial expressiveness, suggesting that highly engaged learners display consistent interaction and clear affective cues.
- **Moderate Engagement**: This group is primarily characterized by Textual indicators (D1_t189, t144), implying that forum discourse—rather than clickstream activity, is a more discriminative signal for identifying learners in the mid-range.

These insights hold practical instructional value. While behavioral logs reliably identify highly engaged learners, monitoring discourse quality appears essential for detecting and supporting students in the moderate engagement category.

### Statistical analysis of model performance consistency

The Friedman test was applied as a non-parametric alternative to repeated-measures ANOVA to assess whether observed performance differences were statistically meaningful. Results (χ^2^ = 4.37, *p* = 0.497) indicated no significant differences in model rankings across folds. Post-hoc pairwise analyses confirmed the absence of systematic divergences between architectures.

Although counterintuitive at first glance, this convergence highlights a central property of the framework: its ability to elevate varied architectures (Transformer, FCN, MCNN) to comparably high levels of performance. Rather than relying on a single model’s architecture, the design of the training protocol, augmentation strategy, and evaluation process shaped consistent outcomes.

For model selection, this finding shifts the decision criterion from accuracy alone to practical considerations. Since predictive differences are statistically indistinguishable, efficiency (Sect. 4.5) becomes decisive. MCNN and TimeCNN, which achieve strong results with lower resource requirements, emerge as preferable for deployment. This statistical consistency ensures that the framework supports flexibility: implementers can choose models based on context and constraints without sacrificing predictive reliability.

### Discrepancy analysis of misclassification patterns and behavioral bias

A post-hoc discrepancy analysis of severe misclassifications by the MCNN model revealed systematic patterns that challenge foundational assumptions in engagement modeling (Table 9). The first pattern, termed “Quiet Achievers,” was identified in three specific instances (Samples 0, 3, and 6) where students with high academic performance were misclassified as having low engagement. Their feature vectors displayed minimal digital footprints; such as near-zero forum posts and neutral facial expressions, indicating that the model penalizes effective but passive learners who do not generate high-frequency interactive signals.

Conversely, the pattern of “Active Strugglers” was observed in two instances (Samples 187 and 201) where students with poor learning outcomes were misclassified as highly engaged. Their profiles showed high volumes of platform interaction, including frequent clicks and logins, which misled the model into interpreting unproductive “busy work” or confusion-driven activity as genuine cognitive investment. These discrepant cases stress a structural vulnerability in activity-centric multimodal frameworks: an over-reliance on behavioral frequency rather than performance quality. Consequently, future frameworks must integrate efficiency metrics, such as performance-to-activity ratios, to better distinguish productive engagement from mere activity. This finding directly corroborates the SHAP analysis in Sect. 4.8, which identified behavioral intensity as a dominant predictor for high engagement; while generally accurate, this dependency exposes the model to error when students generate high-frequency signals without cognitive depth.

**Table 9:** Comparative feature profiles of misclassified student groups.

Student profile	Sample count	Ground truth	Prediction	Behavioral cues (e.g., quiz scores)	Textual activity (e.g., forum posts)	Facial expression
Quiet achievers	3	High	Low	High performance	Negligible/none	Mostly neutral
Active strugglers	2	Low	High	Low performance	High frequency	Mixed/active

### Integrated discussion

Evidence from ablation, sensitivity, and benchmarking analyses indicates that the strength of the framework lies in its overall design rather than in reliance on a single architecture. Temporal data augmentation and heterogeneous ensembling consistently proved essential, each delivering marked improvements in engagement recognition. Their removal caused the sharpest degradations, such as the FCN’s macro F1-score falling from 0.795 to 0.679 without augmentation. Loss function and regularization choices added further stability, with focal loss supporting recognition of minority classes and L2 regularization moderating variance. In contrast, attention layers contributed little, showing that convolutional and dense components already capture the required representational detail.

The stability of these outcomes was confirmed through repeated cross-validation and variation of random seeds. Ensemble accuracy ranged from 0.890 (seed 42) to 0.927 (seed 13), with standard deviations below 0.055. Fold-wise accuracy spanned 0.878 to 0.939. By comparison, the FCN fluctuated between 0.561 and 0.902, showing high sensitivity to data partitioning. Statistical testing reinforced this pattern, with the Friedman test (χ^2^ = 4.37, *p* = 0.497) showing no significant differences among models. This outcome suggests that the protocol does more than raise individual model performance; it reduces variance across architectures, producing a consistent performance plateau independent of design.

Relative to prior studies, the improvement is both methodological and quantitative. Earlier CNN approaches reported 0.88 accuracy with macro F1 as low as 0.61, reflecting persistent difficulty in detecting disengaged students. Even stronger baselines, such as LightGBM (Accuracy = 0.922) and ResNet (Accuracy = 0.917), remain below the 0.976 accuracy and 0.969 macro F1 achieved here under repeated cross-validation. The alignment between accuracy and macro F1 indicates balanced treatment of all engagement states, marking progress from inflated single-split reports to statistically reliable and class-sensitive outcomes.

Efficiency and interpretability analyses extend these findings to deployment. While the ensemble achieved the highest stability, it incurred heavy computational costs. MCNN, by contrast, matched ensemble-level accuracy (0.976) and macro F1 (0.969) with superior efficiency (epoch time ≈ 0.196 s; memory ≈ 111 MB). SHAP analysis of MCNN revealed a structured feature hierarchy, with textual activity dominating but complemented by behavioral and facial features. The consistency of these patterns across the temporal window underscores MCNN’s robustness while offering interpretable outputs that educators can act upon. This dual advantage of efficiency and transparency positions MCNN as the practical deployment choice, with the ensemble providing an upper bound.

The interpretability and stability findings are further contextualized by the Discrepancy Analysis (Sect. 4.9). While SHAP confirmed a feature hierarchy in which behavioral cues emerged as strong predictors, this dependence introduces a structural limitation. A qualitative review of errors showed that the model misclassifies atypical learners, conflating high-frequency activity (e.g., active struggling profiles) with genuine engagement and low-frequency activity (e.g., quiet achievers) with disengagement. These observations indicate that the framework’s high stability must be complemented with fairness-oriented evaluation measures in future iterations to ensure pedagogically sound predictions.

Overall, the framework brings together coherent methodological choices tailored to the demands of educational prediction. Temporal augmentation and class-aware design mitigate imbalance, ensemble modeling improves stability, and the interpretability workflow clarifies the basis of the model’s decisions. The findings indicate that robust and generalizable performance in SEA arises from systematic protocol design rather than architectural novelty. This foundation supports extending the framework to broader educational settings, a direction developed in the concluding section.

## Conclusion and future work

### Summary of contributions

This study introduced a framework for MSEA that integrates repeated cross-validation, temporal data augmentation, class-aware loss functions, and heterogeneous ensembling. Across experiments, the framework surpassed prior baselines, achieving higher accuracy and macro F1-scores while maintaining low variance across seeds and folds. Ablation and sensitivity analyses identified temporal augmentation and ensemble diversity as central drivers of performance, with focal loss and L2 regularization providing additional stability.

Interpretability for the stable framework was conducted on its optimal component, MCNN. This SHAP analysis was deepened to provide feature-level and class-wise attribution, linking specific behavioral and cognitive indicators of engagement to model decisions. The efficiency analysis established MCNN as the optimal practical deployment choice, successfully balancing predictive reliability with superior computational efficiency.

Overall, the study substantiate that the framework’s methodological rigor produces consistent results across distinct model families, establishing a foundation for deployable, reliable, and interpretable educational technologies.

### Practical implications and actionable interventions

A critical challenge in engagement modeling is ensuring that predictive outputs translate into meaningful support for educators and learning systems. The proposed framework addresses this challenge by providing interpretable, stable, and reliable predictions that enable targeted interventions. Robust stability across seeds and folds ensures consistent performance unaffected by specific data partitions or initializations, while efficiency analyses confirm that models such as MCNN and TimeCNN achieve high predictive accuracy with modest computational requirements, facilitating real-time interventions in authentic, large-scale, or resource-constrained learning environments.

This reliable, efficient performance enables two key types of educational support.

1. **Granular and Timely Intervention**: The framework generates fine-grained, real-time data that extends beyond aggregate metrics. Instead of merely reporting low engagement, the model identifies the specific drivers underlying predicted outcomes, enabling immediate, targeted responses:

- **Behavioral Drivers**: Low predictions associated with behavioral indicators (e.g., D2_Behavioral_tXXX such as resource access frequency) suggest technical or environmental interventions, such as automated notifications prompting review of course materials.
- **Textual Drivers**: Low predictions associated with textual indicators (e.g., D1_Textual_tXXX such as insufficient domain-specific vocabulary in forum posts) indicate the need for cognitive or social interventions, such as peer-to-peer discussion prompts or structured feedback on argumentation.

2. **Identifying Atypical Learner Profiles**: The framework’s ability to detect prediction discrepancies (Sect. 4.11) allows the system to identify students whose engagement patterns deviate from conventional assumptions, supporting nuanced, non-punitive interventions:

- **Quiet Achievers**: Students classified as “Quiet Achievers” (High GT / Low Pred) require only classification labeling to prevent unnecessary system notifications, preserving their efficient, self-directed learning approach.
- **Active Strugglers**: Students classified as “Active Strugglers” (Low GT / High Pred) exhibit high behavioral engagement but low learning outcomes, indicating confusion. These learners benefit from individualized guidance or redirection to foundational content, differentiating them from genuinely disengaged peers.

This direct mapping of predictions to targeted interventions transforms the framework from a passive monitoring tool into an active system for personalized educational support, facilitating data-driven resource allocation in authentic learning environments.

### Limitations

The proposed framework and its evaluation are subject to several inherent constraints that define the scope of the findings and outline the boundary conditions under which the results should be interpreted.

- **Data Generalizability and Scope**: A central limitation is the reliance on a single, relatively small dataset (SEA, *N* = 205), which restricts the external validity of the results to similar blended university classroom environments. Although repeated *k*-fold cross-validation, temporal augmentation, and class-aware loss functions were employed to strengthen internal robustness, these procedures cannot substitute for external validation. Broader generalizability requires evaluation across cross-domain contexts (e.g., MOOCs, multilingual cohorts) and diverse learner populations, an essential direction for future research.
- **Modality Constraints and Feature Granularity**.

**Facial modality:** The affective component was limited to three pre-computed emotional states (neutral, happy, surprised). This simplified representation fails to capture the richer spectrum of engagement-relevant affective cues such as confusion, boredom, frustration, or cognitive load variations. Capturing these deeper cognitive-affective states will be crucial for developing more comprehensive engagement models.

**Textual modality:** The textual channel employed TF–IDF vectorization to maintain interpretability and computational feasibility (see Sect. 4.4); however, this approach lacks semantic sensitivity and contextual awareness. Advanced language model embeddings could capture discourse-level signals that TF–IDF cannot represent. Integrating these embeddings is therefore an important step toward improving semantic richness and predictive nuance.

- **Model Complexity and Computational Overhead**: The MCNN architecture shows favourable efficiency, the heterogeneous ensemble, despite providing the highest stability; requires multiple independently operating models. This increases computational overhead and memory footprint, maybe limiting deployment in constrained settings such as mobile learning environments or embedded systems. Reducing this overhead is necessary for real-time, widely accessible deployment.
- **Analysis and Measurement Scope.**

**Ensemble interpretability:** While the heterogeneous ensemble secured the highest predictive stability, computational and technical limitations restricted the SHAP analysis to the best-performing individual component, MCNN. This approach remains justified as the framework’s stability ensures the interpretability analysis is grounded in a reliable performance baseline, a necessary precondition absent in prior unstable studies. Future work should focus on developing scalable attribution methods for complex, multi-model ensembles.

**Temporal and longitudinal assessment:** The proposed framework was not evaluated longitudinally across extended academic terms. Consequently, its capacity to model temporal shifts in engagement such as adaptation, fatigue, or evolving learning strategies remains untested.

**Efficiency metrics:** The Discrepancy Analysis (Sect. 4.11) revealed the need for explicit efficiency-oriented measures (e.g., performance-to-activity ratios) to better disentangle productive engagement (“quiet achievers”) from confusion-driven behaviour (“active strugglers”). This gap represents a structural limitation in the current design and affects the fairness of engagement interpretation.

### Future work

Building on these limitations, the most critical directions for advancing the framework are outlined below:

- Evaluate the framework across cross-domain datasets (e.g., MOOCs, K–12, multilingual cohorts) to establish broader applicability and robustness across diverse learner populations.
- Integrate large language model (LLM) embedding into the textual modality to capture deeper semantic and contextual signals and develop explicit efficiency-based behavioural metrics (e.g., performance-to-activity ratios) to distinguish productive engagement from confusion-driven activity.
- Validate the framework on affect-rich datasets annotated with a broader range of cognitive-affective states such as confusion, frustration, boredom, and focused attention, to more comprehensively model engagement dynamics.
- Reduce computational overhead through lightweight ensemble strategies such as pruning, quantization, and knowledge distillation to support real-time deployment on mobile and embedded devices.
- Conduct longitudinal studies across academic terms to analyse evolving engagement trajectories and enable integration with adaptive learning systems for personalised, real-time intervention.

This study reinforces a broader methodological principle: rigorous evaluation protocols often contribute more to robustness than architectural novelty. By combining temporal augmentation, ensemble diversity, and class-aware objectives, the framework establishes a reliable foundation for deployable, interpretable multimodal engagement analysis in educational contexts.

## Data Availability

The datasets used in this study were derived from publicly available resources. The source data were obtained from the official website of the Ministry of Education of the People’s Republic of China (MOE), and can be accessed without restriction at the following URL: http://www.moe.gov.cn/jyb_sjzl/moe_560/2023.

## Code availability

The custom scripts and algorithms used to process the data and implement the proposed framework will be made available upon reasonable request to the corresponding author, or publicly shared via a persistent repository (e.g., GitHub or Zenodo) following the manuscript’s publication.

## Declarations

### AI declaration

The authors used AI-assisted tools solely for language editing and proofreading to improve clarity and grammar. All scientific content, data analysis, interpretations, and conclusions were developed entirely by the authors.
